# Gene editing using CRISPR/Cas9: implications for dual-use and biosecurity

**DOI:** 10.1007/s13238-017-0493-4

**Published:** 2017-12-04

**Authors:** Diane DiEuliis, James Giordano

**Affiliations:** 1 Center for the Study of Weapons of Mass Destruction, National Defense University, Washington, DC 20057 USA; 2 Departments of Neurology and Biochemistry and Neuroethics Studies Program-Pellegrino Center for Clinical Bioethics, Georgetown University Medical Center, Washington, DC 20057 USA

We have read with interest the recent paper by Kang, et al (2017), addressing clinical and ethical issues related to the safe and responsible use of CRISPR/Cas. The authors provide a number of important considerations about the current capabilities offered by this novel gene-editing tool, including germ line editing in embryos, and potential diverse uses in adult human applications. The authors posit that the tool in of itself does not represent a threat, and that periodic assessment will ensure its responsible use.

We agree, but with caveat: namely, that any tool that imparts great capability also involves at least some risk, if not threat, that the power conferred by such capacity can be used to leverage or evoke a variety of ends. This deviation of intent is a principal concern of dual-use research and its applications. Apropos such potential, we provide a complementary view that considers capricious, if not nefarious use of CRISPR to modify biological entities (e.g., microbes, plants, animals, and humans) and/or produce bioagents that pose risk and/or threat to public safety (DiEuliis and Giordano, 2017). We view this as a real, present and certainly near-term future concern, and propose a set of additional considerations that we feel are important security components that will be essential to assessing the use of CRISPR/Cas.

We propose that agreed-upon international, ethical “norms” for human modification for therapeutic purposes are relevant and applicable to any such use of this technique. Kang et al (2017) advocate international standards of ethics, and note efforts made to date by the National Academies (2017) in this regard. We concur with the need for international ethical standards and guidelines, and also note the need for more engaged discourse to define the needs, values and ethical system(s) and principles to be employed (Palchik, Chen, and Giordano, 2017; Lanzilao, Shook, Benedikter, and Giordano, 2013) Furthermore, in recognition of the potential risk/threat posed by genetic modification, we strongly endorse involvement of the Biological Toxins and Weapons Convention (BTWC), in order to ensure inclusion of biosafety and biosecurity communities in any such deliberation and determination of standards. Templates may exist and could be consulted for the development of international norms and best practices through engagement of expertise in technical aspects of emergent technology and security fields (Talinn Manual, NATO, Carnegie Endowment 2017). Expanding the scope and platform of international dialogue can be instrumental to ensuring that all aspects of emerging biotechnological tools and methods are evaluated for their potential to be weaponized or used in other ways that threaten public safety (Gerstein and Giordano, 2017).

Importantly, many of the agents modified or designed for therapeutics will have dual uses of concern. A particularly good example are those that are able to modify human biological, cognitive and/or behavioral function(s). A recent study of US public attitudes toward genetic enhancement showed that while the use of CRISPR for therapeutics was viewed as being appropriate, its use for bio-enhancement was regarded as far less acceptable (Scheufele et al., 2017). But public attitudes often do not reflect the needs and values desiderata of the military, and CRISPR and related techniques offer considerable potential to both create novel weaponizable entities, and to modify aspects of human performance in ways that are relevant and meaningful to warfare operations. In considering implications on a global scale, countries could embed these genetic modification programs within academic and/or commercial research and development infrastructures to make dual-use applications difficult to detect (Giordano, 2016). As well, the relative availability of this technique enable increasing use by public research and do-it-yourself (i.e., biohacking) communities which could foster risk incurred by both inadvertent misuse and/or intentional development of products that threaten public safety (Giordano, 2017). These dual use aspects should be included in critical discussions.

Uses of CRISPR in contexts outside of human biology—to modify plants, agricultural products, animals, and insects, prompted recent review of the Coordinated Framework for the Regulation of Biotechnology (National Academies, 2017), which concluded that synthetic biology will innovate a much wider number of products requiring regulatory oversight and governance. In addition, new commercial enterprises and lowered barriers to market entry may alter the number and types of regulated entities to blur jurisdictional concepts of “product sponsor,” “product developer,” or “manufacturer”, and obfuscate responsibilities for development and use.

But techniques such as CRISPR need not be only regarded for their dual use potential. To be sure, gene editing can directly affect the traditional bioweapons arena. There have been discussions of the potential for CRISPR to be used to create or further manipulate viruses, bacteria and bacterially produced toxins (i.e., select agents) (Clapper, 2016). Previous bioweapons programs focused on molecular experimentation with select agents to make them more weaponizable. Now, techniques such as CRISPR can both enable improved capacity to manipulate pathogens for this purpose, and may make such manipulations easier and quicker to accomplish. As well, CRISPR-based approaches could be used to incur disease by inducing harmful genetic modifications *in vivo*. Just as CRISPR kits can be made to order from industry providers, means of directly delivering CRISPR to incur genetic modifications are also being developed (Mullin, 2017). Nefarious actors using these techniques would not care about “off target” effects, as long as the agent achieves disruptive or destructive effect(s). In such cases, simply engineering a molecule capable of producing harmful modification to human DNA might provide a viable (and none too difficult) biothreat option.

In conclusion, we argue that further discussion is needed to evaluate both the scope and extent of current capabilities—and the challenges and opportunities to further develop and/or exploit such techniques—to be used in ways that may impact public safety and health. For as science and technology advance, there will be need to iteratively re-assess current perspectives and definitions of what constitutes weaponizable biology, and to engage international discourse toward more effective, efficient guidance and governance of these agents and the activities and entities that are capable of producing them.
